# Lack of Healthy Food in Small-Size to Mid-Size Retailers Participating in the Supplemental Nutrition Assistance Program, Minneapolis–St. Paul, Minnesota, 2014

**DOI:** 10.5888/pcd12.150171

**Published:** 2015-08-27

**Authors:** Melissa N. Laska, Caitlin E. Caspi, Jennifer E. Pelletier, Robin Friebur, Lisa J. Harnack

**Affiliations:** Author Affiliations: Caitlin E. Caspi, University of Minnesota, School of Medicine, Department of Family Medicine and Community Health, Minneapolis, Minnesota; Jennifer E. Pelletier, Robin Friebur, Lisa J. Harnack, University of Minnesota, School of Public Health, Division of Epidemiology and Community Health, Minneapolis, Minnesota.

## Abstract

**Introduction:**

The US Department of Agriculture has stocking criteria for healthy foods among Supplemental Nutrition Assistant Program (SNAP)-authorized retailers. Increased access to healthy food could improve diet quality among SNAP participants, which has implications for chronic disease prevention. The objective of this study was to quantify healthy foods stocked in small-size to mid-size retailers who are authorized under SNAP but not under the Special Supplemental Nutrition Program for Women, Infants, and Children (WIC).

**Methods:**

We used formative, cross-sectional data from a large policy evaluation to conduct secondary analyses. Store audits were conducted in 2014 in 91 randomly selected, licensed food stores in Minneapolis and St. Paul, Minnesota. Supermarkets and retailers participating in WIC, which are required to stock healthy foods, were excluded as were other stores not reasonably expected to stock staple foods, such as specialty stores or produce stands. Availability of milk, fruits, vegetables, and whole-grain–rich foods was assessed.

**Results:**

The 91 stores studied were corner stores, food–gas marts, dollar stores, and pharmacies. More than half carried 1 or more varieties of fat-free or low-fat milk, fresh or canned fruit, and whole-grain–rich cereal. However, only one-third stocked 1 or more varieties of fresh vegetables and only one-quarter stocked whole-grain–rich products, such as whole-grain-rich bread (26%) or tortillas (21%) or brown rice (25%). Few stores stocked at least 2 varieties of each product.

**Conclusions:**

Many stores did not stock a variety of healthy foods. The US Department of Agriculture should change policies to improve minimum stocking requirements for SNAP-authorized retailers.

## Introduction

Many low-income communities have limited access to healthy foods (1). Previous research indicates that supermarkets are more likely to locate in high-income and low-minority areas, and smaller stores (eg, convenience stores) are more likely to locate in low-income and high-minority areas (1). Disparities in food access may contribute to health disparities, because better access to supermarkets and healthy foods is associated with healthy diets and reduced risk of obesity (2), and access to convenience stores is associated with increased health risks (1,3–6). Supermarkets offer a wider variety of healthy, high-quality foods than smaller stores, which tend to carry high-calorie, processed foods (1,7).

Policy action to improve access to healthy foods has been identified as a strategy for obesity prevention. Given that the Supplemental Nutrition Assistance Program (SNAP) provides nutrition assistance to 42 million low-income Americans each month (8), finding mechanisms to improve the dietary patterns of SNAP participants through SNAP policy change has been a topic of much attention (9,10). Strategies have focused primarily on consumer-level approaches, such as consumer education and providing incentives for healthy purchases (11).

Complementary store-based approaches have received less emphasis. For example, unlike the Special Supplemental Nutrition Program for Women, Infants, and Children (WIC), SNAP regulations do not require vendors to stock healthy foods, but require that vendors offer 3 varieties of 4 categories of staple foods (ie, meat, poultry or fish, bread or cereal, vegetables or fruits, dairy products) with perishable foods in 2 of these categories. Thus, a vendor could meet these requirements by stocking foods such as high-fat meats, white bread, and ice cream. Vendors are also considered to meet these standards if at least half of store sales are from staple foods.

The Agricultural Act of 2014 (ie, the 2014 Farm Bill) mandated alterations to these stocking requirements, requiring stores to carry 7 items across the 4 categories of staples, including perishable items in 3 categories (12). This alteration will now undergo US Department of Agriculture (USDA) rulemaking. Furthermore, USDA has the authority to add specificity to these requirements to include a focus on healthy foods, such as fruits, vegetables, low-fat dairy products, and whole-grain–rich products.

This rulemaking could have an effect on a wide array of food outlets. Nationally, 20% of SNAP transactions occur in small stores, such as convenience stores or other small food stores, where availability of healthy foods may be limited (13). Benefit redemptions at small stores are more frequent in areas with poor health outcomes, such as low-income and nonmetropolitan areas, as well as among single-adult and minority households (13,14). Furthermore, previous research has shown that purchases from convenience stores in particular tend to consist of calorie-dense, prepackaged products of poor nutritional quality (15,16).

The purpose of this study was to describe healthy food availability among small-size to mid-size, SNAP-authorized food retailers that were not WIC-authorized.

## Methods

This secondary analysis used formative, prebaseline data collected for a larger policy evaluation (R01-DK104348–01A1), the Staple Food Ordinance Evaluation (STORE) Study examining the impact of a local policy change on healthy food access, particularly in small-size to mid-size food stores. Stores were randomly selected for study inclusion in the STORE study from lists of licensed grocery retailers (n = 841) obtained from the Minneapolis Health Department, which regulates Minneapolis retail licensing, and the Minnesota Department of Agriculture, which regulates St. Paul licensing. The STORE study focused on licensed stores outside the 2 downtown commercial districts that had the greatest potential to improve healthy food availability, including convenience stores and food–gas marts. Stores were ineligible for the prebaseline assessment in the STORE study if they 1) were supermarkets (n = 16), 2) were identified as WIC-authorized by using state-level data (n = 175), 3) had pre-identified address problems (ie, mailed letters were returned) (n = 15), or 4), or were not expected to stock an array of staple foods, for example, liquor stores, specialty shops (eg, stores that primarily sold spices, olive oil, coffee and baked goods), small market vendors (eg, meat or produce vendors, imported goods vendors selling a limited range of products), and stores with 100 square feet or less of retail space (n = 325).

Of the 310 eligible stores, 172 were visited in winter and spring 2014 to meet the target data collection of 120 or more stores for the prebaseline assessment period for the STORE study. Of these, 13 refused participation, 20 were newly WIC-authorized, 3 did not sell food, 1 was under renovation, 6 were out of business, and 10 could not be located. Additionally, because the larger study assessed stores regardless of SNAP participation status, 26 stores assessed were not SNAP-authorized, and 2 had incomplete SNAP data; these were excluded from the present analyses. The final analytic sample was n = 91.

### Data collection

Store audits were conducted on weekdays between 9:45 AM and 4:30 PM. In teams of 2, data collectors entered stores, identified themselves, and asked for permission to conduct the audit. All stores invited to participate received a mailed letter in advance describing the study. The Institutional Review Board of the University of Minnesota approved all study protocols.

### Store audit

The store audit was based on a tool developed at the Yale Rudd Center for Food Policy and Obesity and was previously used to evaluate the impact of 2009 WIC policy revisions in small stores (17). It is a standardized inventory adapted from the Nutrition Environment Measure Survey in Stores tool and has demonstrated good inter-rater and test–retest reliability (5,17). We adapted the Rudd Center tool by substituting WIC-approved items (eg, frozen dinners) for some non-WIC–approved foods (eg, eggs and canned fish). The Rudd Center tool was slightly modified further to align with local WIC requirements. The modified tool included specific product types, such as plain shredded cheese in containers 8 ounces or larger (which is WIC-allowable in Minnesota, but not in Connecticut, where the assessment tool was originally developed) and other store features of interest (eg, use of a point-of-sale cash register system). The inventory measured availability of 69 specific items and the quality of 20 designated fruits and vegetables. It also measured varieties and amounts of milk (in half-gallon or 1 gallon containers only); frozen, canned, and fresh fruits and vegetables (with no added ingredients other than salt in canned products); whole-grain–rich bread (ie, whole grains listed as the first ingredient); whole wheat or corn tortillas; brown rice; and whole-grain–rich cereals (in packages ≥12 oz).

Data collectors assessed the varieties and total amounts (number of items, weight) of all fresh fruits and vegetables. To estimate total weight of items sold individually (eg, apples) the count of each variety was multiplied by a standard item weight (eg, 1 medium banana = 0.41 lb). Standard weights were based on USDA-reported averages, with refuse weight added (18). For items not sold individually (eg, baby carrots) data collectors recorded package weights. Where no USDA data or package weight was available (eg, garlic) (n = 7), research staff visited a local supermarket and weighed a sample of the items to estimate the average weight per item.

Quality of 20 common types of fruits and vegetables was rated on the basis of the percentage of each type of food that was molded, wrinkled, shriveled, bruised, or wilted: excellent (A) = 0%; very good (A−) = 1% to 10%; good (B) = 11% to 24%; fair (B−) = 25% to 50%, and poor (C) = 51% or more.

Availability of whole-grain–rich foods was assessed as 1) whole-grain–rich cereals, bread, and tortillas; 2) brown rice; and 3) other whole grains (eg, plain uncooked oats or oatmeal, whole cornmeal, unpopped popcorn, whole-wheat flour, teff flour, quinoa), excluding tortilla chips and pre-popped popcorn; and other whole-grain–rich products (eg, pasta, injera). Whole-grain–rich products were identified by data collectors by examining product ingredient lists and including those for which the first ingredient was whole grain.

Interrater reliability was assessed for 33 stores. Agreement for 61 of 69 items was excellent (percentage agreement = 0.91–1.00) and good for an additional 8 items (percentage agreement = 0.82–0.88), including regular fat cheddar cheese, 100% juice, canned peas and beans, white bread, and dry lentils. Overall agreement was good (0.86, comparing grades of A/A−, B/B− and C) for produce quality scores.

### Analyses

We examined store characteristics (eg, store type, number of cash registers) and food availability using descriptive statistics. To create categories, composite variables were calculated for low-fat and fat free milk, including skim, 1%, and unflavored soy milk; and vegetable subtypes (ie, dark green vegetables [eg, broccoli, bok choy, kale], red and orange vegetables [eg, carrots, tomatoes, acorn squash], starchy vegetables [eg, corn, plantains, potatoes], and other vegetables [eg, celery, cucumbers, onions]). These variables were based on classifications in *Dietary Guidelines for Americans* (19).

We summarized availability and median quality of the 5 most commonly stocked types of fresh fruits and vegetables. Overall fruit and vegetable quality scores for were computed on the basis of 20 varieties for which data were collected. Analyses were conducted in Stata, version 13.1 (StataCorp LP).

## Results

Most SNAP-participating stores in the final analytic sample were corner stores or small groceries (34%) or food–gas marts (43%); fewer were dollar stores (10%) or pharmacies (13%). Fifty-nine percent of stores had 6 or fewer aisles, and 64% had 2 or fewer cash registers (Table 1).

**Table 1:** Characteristics of Study Sample of Small-Size to Mid-Size Food Stores (N = 91)^a^ Participating in the Supplemental Nutrition Assistance Program, Minneapolis–St. Paul, Minnesota, 2014

Store Characteristics	Number	Percentage
**Store type**		
Corner or small grocery	31	34
Food–gas mart	39	43
Dollar store	9	10
Pharmacy	12	13
**Number of aisles**		
1–2	11	12
3–6	42	47
7–10	37	41
**≤2 cash registers**	58	64
**Open 24 h daily**	13	14
**Point-of-sale cash register system**	54	62
**Sells tobacco**	69	76

a Sample does not include stores participating in the Special Supplemental Nutrition Program for Women, Infants, and Children (WIC).

Most stores offered some quantity of fat-free or low-fat milk (64%), fresh fruits (62%), canned fruits or vegetables (93%), or whole-grain–rich cereal (80%) (Table 2). Fewer than one-third of stores (31%) stocked fresh vegetables, with fewer stocking nutrient-rich varieties, such as dark green vegetables (9%) or red and orange (20%) vegetables. Approximately 1 in 4 stores carried frozen fruits or vegetables, whole-grain–rich bread and tortillas, and brown rice.

**Table 2:** Availability of Healthy Foods and Beverages in Study Sample of Small-Size to Mid-Size Food Stores (N = 91)^a^ Participating in the Supplemental Nutrition Assistance Program, Minneapolis–St Paul, Minnesota, 2014

Category	Any Available^a^, %	≥2 Varieties Available, %	≥3 Varieties Available, %
**Dairy**			
Skim or 1% cow’s milk or unflavored soy milk	64	56	2
**Fruits and vegetables**			
Fresh fruit	62	54	43
Fresh vegetables, any	31	26	22
Fresh vegetables, dark green^c^	9	4	1
Fresh vegetables, red and orange^d^	20	14	7
Fresh vegetables, starchy^e^	19	2	0
Fresh vegetables, other^f^	30	16	9
Canned fruits or vegetables	93	78	71
Frozen fruits or vegetables	23	20	19
**Whole-grain–rich products**			
Whole-grain–rich bread	26	12	3
Whole wheat or corn tortilla	21	3	2
Brown rice	25	3	1
Whole-grain–rich cereal	80	74	60

a Sample does not include stores participating in the Special Supplemental Nutrition Program for Women, Infants, and Children (WIC) program.

b Stocked at least 1 item per category.

c Dark green vegetables include broccoli, bok choy, chard, collards, and kale (19).

d Red and orange vegetables include whole and baby carrots, tomatoes, red peppers, chili peppers, acorn squash, and yams (19).

e Starchy vegetables include corn, plantains, jicama, and potatoes (19).

f Other vegetables are cabbage, celery, cucumber, onion, green peppers, artichokes, beets, red cabbage, cauliflower, eggplant, rutabaga, sprouts, zucchini, turnips, and yellow squash (19).

Few stores carried multiple varieties of healthy products. One-quarter (26%) stocked 2 or more varieties of fresh vegetables; fewer (2%–16%) carried multiple varieties of each vegetable subtype. Most stores that carried frozen fruits and vegetables stocked multiple varieties; fewer stores stocked 2 or more varieties of whole-grain–rich bread (12%), tortillas (3%), or brown rice (3%). The median weight of fresh produce available in stores was 13 pounds, the equivalent weight of 10 apples plus 10 oranges plus 10 bananas of medium size.

Quality scores for fresh produce primarily were median ratings of A, the highest rating possible (Table 3); the median quality score for 2 produce types (bananas and lettuce) received median ratings of A−. Most stores received scores of A or A− for all the fresh fruit (58% of stores) or vegetables (62%) they carried (Table 4). Few store received poor ratings.

**Table 3:** Quality of Common Fresh Fruits and Vegetables Sold in Study Sample of Small-Size to Mid-Size Food Stores (N = 91) Participating in the Supplemental Nutrition Assistance Program, Minneapolis–St. Paul, Minnesota, 2014

Most Commonly Stocked Item	Stores at Which Item Type Was Available, %	Median Quality Score^a^
**Fruits**		
Banana	47	A–
Apple	45	A
Orange	41	A
Lime	14	—b
Lemon	13	A
**Vegetables**		
Onion	27	A
Tomato	16	A
Potato	14	—b
Celery	10	—b
Lettuce	10	A–

Abbreviation: —, not available.

a Quality scores were rated on the basis of the percentage of each type of food that was molded, wrinkled, shriveled, bruised, or wilted: excellent (A) = 0%; very good (A−) = 1% to10%; good (B) = 11% to 24%; fair (B−) = 25% to 50%, and poor (C) = 51% or more.

b Quality data were collected on only 20 selected fruits and vegetables. Scores were not available for limes and potatoes because quality for these was not assessed. Scores for celery were suppressed because of missing data and a small sample size.

**Table 4:** Overall Quality of Common Fresh Fruits and Vegetables Sold in Study Sample of Small-Size to Mid-Size Food Stores (N = 91) Participating in the Supplemental Nutrition Assistance Program, Minneapolis–St. Paul, Minnesota, 2014

Overall Store Rating	No. (%) of Stores^a^
**Fruit quality^b^**	
All A or A–	32 (58)
A and B	17 (31)
At least one C	6 (11)
**Total**	55 (100)
**Vegetable quality^b^**	
All A or A–	16 (62)
A and B	3 (12)
At least one C	7 (27)
**Total**	26 (100)

a Quality data were collected on only 20 selected fruits and vegetables. Sample sizes for overall quality scores do not total 91 because they refer only to those stores that stocked 1 or more of the 20 varieties of fruits or vegetables for which data on quality were collected.

b Quality scores were rated on the basis of the percentage of each type of food that was molded, wrinkled, shriveled, bruised, or wilted: excellent (A) = 0%; very good (A−) = 1% to10%; good (B) = 11% to 24%; fair (B−) = 25% to 50%, and poor (C) = 51% or more.

## Discussion

Findings from this study indicate that a substantial number of small-size to mid-size food stores that participate in SNAP, but not WIC, in Minneapolis and St. Paul, Minnesota, did not carry a variety of healthy foods, such as fresh or frozen vegetables, and whole-grain–rich foods, such as bread, tortillas, and brown rice. Most stores offered limited varieties of most healthy items, except canned fruits and vegetables and whole-grain–rich cereals.

Reducing health disparities through systems-level policy and environmental change has become a major focus of obesity prevention and nutrition promotion research and practice. To this end, an increasing number of calls have been issued to improve healthy food choices among SNAP participants across the United States as a means of health promotion and chronic disease prevention (9,10). Numerous individual-level and store-level strategies have been proposed, such as providing additional SNAP benefits and rewards for healthy food purchases, prohibiting the purchase of unhealthy foods with SNAP benefits, and nudging participants toward healthy SNAP purchasing in other ways (9). Although we do not know if increasing minimum stocking standards for retailers by itself would improve the nutritional quality of food purchasing among participants enrolled in the SNAP program, such a policy change could be a valuable complement to other strategies being proposed. Results from evaluations of policy changes in 2009 in the WIC program indicated that increasing healthy food availability in participating stores, in combination with increasing participant vouchers for healthy foods and implementing other supporting change in the program, resulted in numerous positive outcomes, such as healthier food purchasing, more favorable dietary patterns, and even potential shifts in weight status among participants (20–24).

Increasing minimum stocking standards for retailers would be expected to have an impact on only a limited subset of SNAP participants and SNAP-related purchases. For example, 64% of SNAP benefits nationwide are redeemed at supermarkets and supercenters (13), which are stores that would be unaffected by increases in stocking standards. However, research indicates that smaller retailers receive a larger percentage of SNAP benefit redemptions in low-income urban areas compared with high-income suburban areas. For example, a 2014 study in Minneapolis and St. Paul showed that the percentage of benefits redeemed at convenience stores was nearly 4 times higher in the 8 neighborhoods with the greatest number of SNAP clients compared with redemption rates for convenience stores in neighborhoods with fewer SNAP clients (15% vs 4% of SNAP benefits redeemed) (14). Thus, although most SNAP benefits are redeemed in supermarkets or supercenters, which typically offer a wide array of healthy foods and beverages for sale, the neighborhoods in greatest need would likely be those most affected by improvements in minimum stocking standards. Therefore, an increase in stocking standards could help support more comprehensive efforts to address health-related disparities in these communities, albeit in a limited way.

Other implementation issues to consider include retailer burden and technical assistance needs. For example, stocking perishable items, including fresh produce and whole-grain–rich bread, may present specific challenges for store owners (25). Owners of smaller stores have reported difficulty in identifying distributors to deliver perishable products with adequate frequency and in sufficiently small quantities at a reasonable cost. However, SNAP policies mandating participating retailers to carry these types of healthy products would open a new market for distributors, possibly making it profitable to supply a large number of small stores with these products. Furthermore, owners and their staff may need training on produce handling, storage, and merchandizing to extend shelf life and improve sales (26). Adequate infrastructure for storage and display of perishable products, such as refrigerators or coolers, is another issue to address (26). If SNAP retailers are mandated to stock a minimum supply of perishable products, USDA should consider making technical assistance available to retailers. Additional efforts may be needed to connect these retailers with low-interest loans or other sources of funding that can support infrastructure enhancements (27).

Despite these challenges, our findings indicate that among the small-size to mid-size stores in our sample that stocked fresh produce, produce quality was good. Most stores exclusively received ratings of A and A− on the most common varieties of produce. A fear often cited in local discussions around healthy foods in small food stores is that the quality of perishable items will be unacceptable to customers. Our data suggest that there are existing business models for stocking high-quality produce in these stores. More data are needed to understand the factors enabling these businesses to maintain such a high quality.

To our knowledge, this study presents some of the first evidence documenting healthy food availability among small-size to mid-size SNAP retailers that do not also participate in WIC. Other work has more broadly documented the lack of healthy food availability and unhealthy purchasing in small food retail outlets overall (1,15,16); collectively, this body of literature can help inform USDA as changes to SNAP minimum stocking requirements are considered.

Despite its contribution to the literature, several limitations of this study should be noted. First, these data were collected from a specific geographic region in Minnesota, limiting generalizability. The study was also limited by need for parsimony in data collection, which omitted some foods otherwise considered healthy (eg, dried fruit), and a limited study sample that did not include SNAP-authorized stores in downtown commercial districts. In addition, data on other factors, such as food and beverage pricing and stakeholder perspectives on opportunities for SNAP policy change, were not included here and should be incorporated in future research. Previous research with key government and public health stakeholders has indicated that major barriers to improving dietary intake among SNAP participants include various environmental factors, such as limited access and high cost of healthy foods, as well as factors such as the marketing of unhealthy food in low-income communities and stressors associated with living in poverty (28,29). It is also important to better understand and carefully consider the key stakeholder perspectives of SNAP-participating retailers and SNAP participants themselves as USDA and others develop strategies to address each of these challenges.

Our results underscore the limited availability of healthy foods in small-size to mid-size food retailers. USDA should consider requiring SNAP-authorized retailers to carry minimum quantities of healthy foods, including vegetables (particularly dark green and red and orange vegetables) and whole-grain–rich foods, as part of a comprehensive approach to nutrition promotion within the SNAP program. Perspectives of local, state, and national stakeholders should be considered when establishing these requirements, and store-level technical assistance and other supports (26) would be needed to assist retailers in meeting requirements. To further ensure store-level success, other strategies promoting healthy SNAP purchasing and driving customer demand may also be needed.
